# Letter to the editor regarding “Is minimally invasive superior than open transforaminal lumbar interbody fusion for single-level degenerative lumbar diseases: a meta-analysis”

**DOI:** 10.1186/s13018-019-1196-8

**Published:** 2019-05-29

**Authors:** Larry E. Miller

**Affiliations:** Miller Scientific Consulting, Inc., 1854 Hendersonville Road, Asheville, NC 28803 USA

To the editor,

The meta-analysis by Li et al. [1] published in the journal evaluated randomized controlled trials (RCTs) comparing minimally invasive versus open approaches to transforaminal lumbar interbody fusion (TLIF). No known meta-analysis on this topic has included only results from RCTs, and therefore, this would represent the highest level of evidence available. Yet upon review of this article, there are serious methodological issues that nullify the conclusions.

The authors state that 7 RCTs were included in this review. Yet only 2 of the 7 studies were actually RCTs—the studies of Serban et al. [2] and Wang et al. [3] The remaining 5 studies utilized prospective or retrospective nonrandomized comparisons [4–8]. Among the nonrandomized studies that were incorrectly included, Table 1 includes samples of text that clearly identifies each as a nonrandomized study. This is especially concerning since the authors reported that duplicate verification of study eligibility was performed. Further, in Figs. 2 and 3 (Li et al. [1]) in their meta-analysis, they state that 5 of 7 studies had low risk of bias as it relates to random sequence generation. Clearly, the authors have made egregious errors in the classification of the included studies.

**Table 1:** Listing of nonrandomized studies described as randomized controlled trials and locations of study design descriptions within each manuscript

Study	Abstract	Manuscript text
Singh et al. [4]	Study design/setting: “This study was a nonrandomized, nonblinded prospective review.”	Patient selection: “We performed a retrospective analysis of…”
Kulkarni et al. [5]	–	Materials and methods: “The patients were given the option to decide between MI-TLIF and O-TLIF, the cost of the procedure was a single major deciding factor.”
		Discussion: “First, it is a nonrandomized study as the patients were given an option to choose the procedure. A randomized study will provide more convincing, evidence-based results.”
Lee et al. [6]	Study design: “Prospective observational cohort study.”	Materials and methods: “The patients were not pre-selected for either group; the type of operation undertaken was based on surgeon’s and patient’s preferences.”
		Discussion: “Firstly, it is an observational cohort comparison study and not a randomized controlled trial…”
Seng et al. [7]	Study design: “Retrospective analysis of prospectively collected data.”	Materials and methods: “This was a matched-pair analysis of patients with prospectively collected data…”
		Materials and methods: “The patients were not randomized to the type of surgical procedure, and the decision to perform MIS or open TLIF was surgeon dependent.”
		Discussion: “First this is a matched-pair analysis and not a randomized prospective study. The patients were not randomized to the type of surgery, and the decision to perform open or MIS TLIF was surgeon dependent.”
Wang et al. [8]	Study design: “This is a prospective single-center nonrandomized control clinical study…”	Discussion: “There are several limitations in the present study. A randomized controlled trial should be considered to provide convincible evidence-based conclusions in the future.”

In Fig. 5 (Li et al. [1]) of their meta-analysis, another major error presents itself. It is noted in the forest plot that hospital stay was 2.2 days longer with minimally invasive TLIF in the Serban study [2]. Yet, hospital stay was actually 2.2 days shorter with minimally invasive TLIF in this study. This additional error fundamentally impacts the meta-analysis results since reporting of correct data would have led to a different conclusion—that minimally invasive TLIF is associated with a shorter hospital stay relative to open TLIF.

Given the major and obvious flaws in study selection and data analysis and consequently, the potential risk of other less obvious deficiencies, the authors and editors are encouraged to retract this article.

## Data Availability

Data sharing not applicable to this article as no datasets were generated or analyzed during the current study.
